# Supplementary material for: Predicting visual function by interpreting a neuronal wiring diagram
Source: Nature. 2024 Oct 2;634(8032):113–23. doi: 10.1038/s41586-024-07953-5 (PMC11446822; doi:10.1038/s41586-024-07953-5)
Supplement: Supplementary file 6 — CRF and ERF predictions for individual TmY4 and TmY9 cells. Analogous to Supplementary Data 3, but for TmY target types. Shown are the top four monosynaptic pathways, the strongest pathway passing through each of the top ten intermediary types (ranking from Extended Data Fig. 7), and the trisynaptic pathway Tm1–TmY–Dm3–TmY (see the section entitled Prediction of spatial normalization). [file 41586_2024_7953_MOESM6_ESM.zip › DataS4/TmY9qΓèÑ/720575940639019327.pdf]

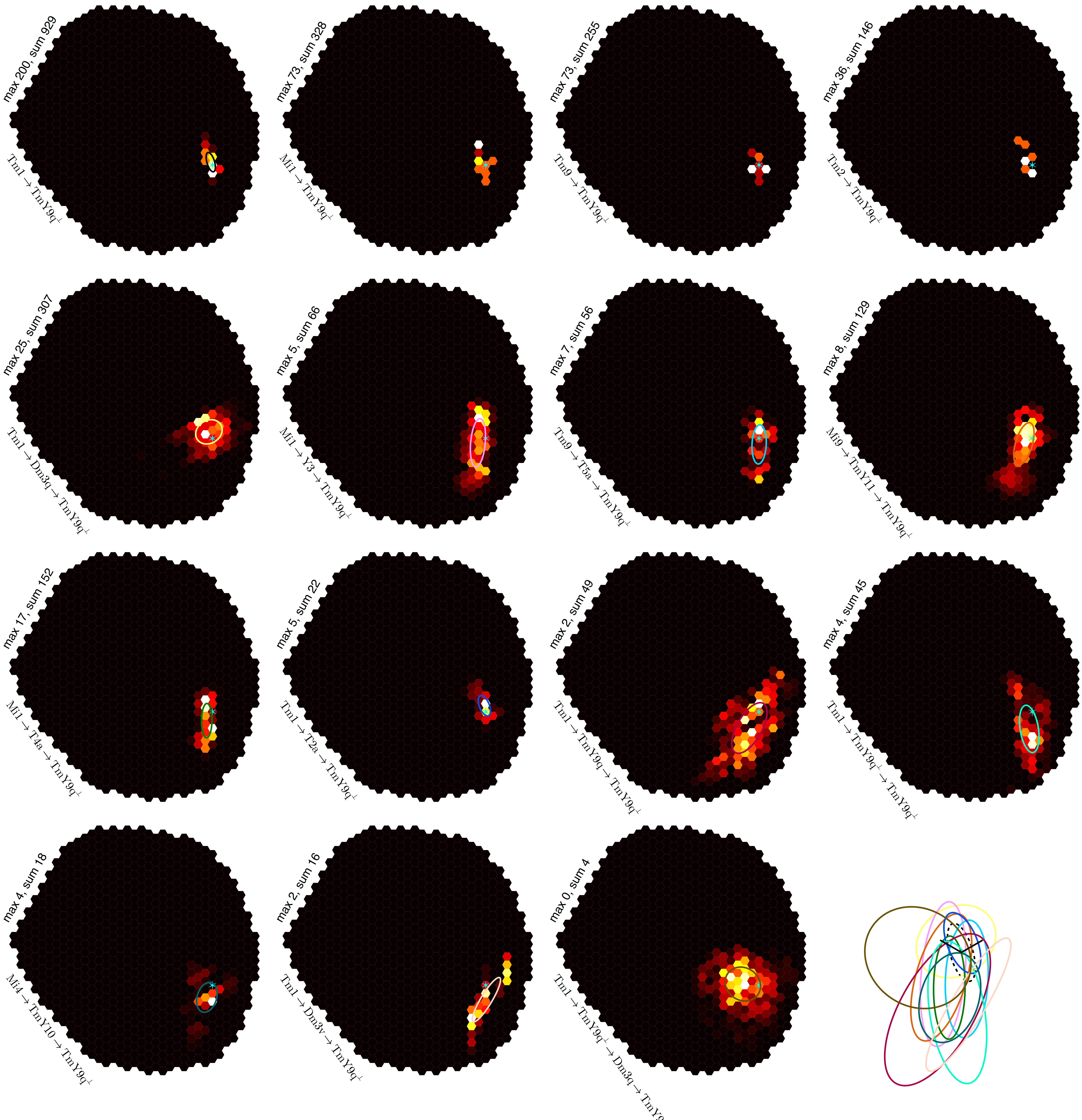
max 200, sum 929
Tm1 → TmY9q⊥
max 73, sum 328
Mi1 → TmY9q⊥
max 73, sum 255
Tm9 → TmY9q⊥
max 36, sum 146
Tm2 → TmY9q⊥
max 25, sum 307
Tm1 → Dm3q → TmY9q⊥
max 5, sum 66
Mi1 → Y3 → TmY9q⊥
max 7, sum 56
Tm9 → T5a → TmY9q⊥
max 8, sum 129
Mi9 → TmY11 → TmY9q⊥
max 17, sum 152
Mi1 → T4a → TmY9q⊥
max 5, sum 22
Tm1 → T2a → TmY9q⊥
max 2, sum 49
Tm1 → TmY9q → TmY9q⊥
max 4, sum 45
Tm1 → TmY9q⊥ → TmY9q⊥
max 4, sum 18
Mi4 → TmY10 → TmY9q⊥
max 2, sum 16
Tm1 → Dm3v → TmY9q⊥
max 0, sum 4
Tm1 → TmY9q⊥ → Dm3q → TmY9q⊥
